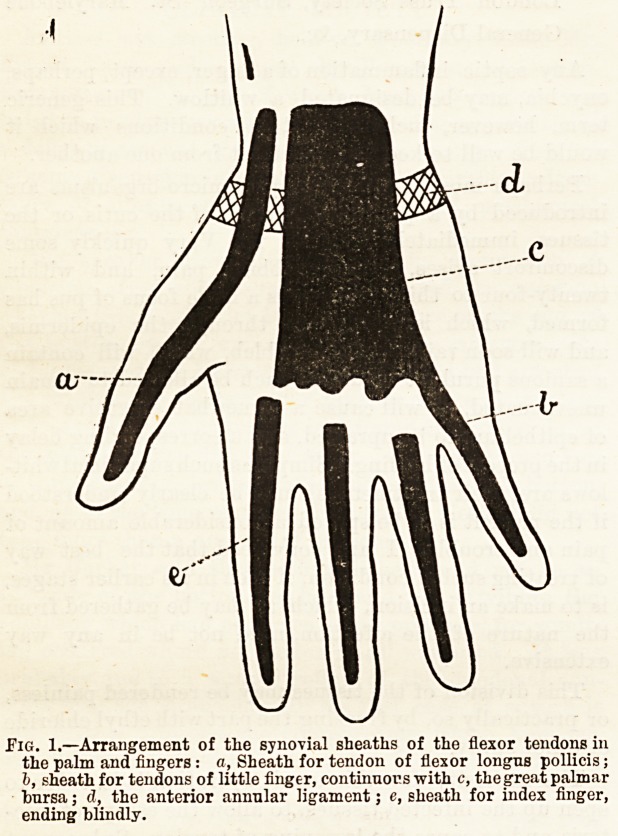# Whitlow

**Published:** 1897-02-20

**Authors:** W. McAdam Eccles

**Affiliations:** Assistant Surgeon West London Hospital, City of London Truss Society, Surgeon St. Marylebone General Dispensary, &c.


### II.—WHITLOW.

By W. McAdam Eccles, M.S. (Lond.), F.R.C.S. (Eng.), Assistant Surgeon West London Hospital, City of London Truss Society, Surgeon St. Marylebone General Dispensary, &c.

Any septic inflammation of a finger, except, perhaps, onychia, may be designated a whitlow. This generic term, however, includes certain conditions which it would be well to keep very distinct from one another.

Perhaps most commonly septic micro-organisms are introduced by a punctured wound of the cutis or the tissues immediately beneath it. Very quickly some discomfort arises, then throbbing pain, and within twenty-four to thirty-six hours a little focus of pus has formed, which is easily seen through the epidermis, and will soon raise this into a bleb, which will contain a sanious purulent fluid. If such be allowed to remain unevacuated, it will cause a somewhat extensive area of epithelium to be upraised, and a corresponding delay in the process of healing. Simple as such superficial whitlows are, their treatment should be clearly understood if the patient is to be spared a considerable amount of pain and trouble. I am convinced that the best way of treating such a condition, if seen in its earlier stages, is to make an incision, which, as may be gathered from the nature of the affection, need not be in any way extensive.

This division of the tissues may be rendered painless, or practically so, by freezing the part with ethyl chloride or ether spray.

The necessity for this small operation is in order to open up the infected tissues, to allow the escape of bacteria and to cause the lessening of tension. Subsequent immersing the finger for some length of time in very hot 1-4,000 perchloride of mercury solution will greatly relieve the pain, and promote resolution. In this way spread of the inflammation will be arrested, and destruction of tissue avoided.

If the inflammation has commenced in or extended more deeply into the fat and connective tissue of the finger, there will be much tense swelling, exquisite tenderness, and severe throbbing pain.

These are the cases which have to be differentiated from the condition in which the sheath of the flexor tendons is involved. This form of whitlow is probably by far the most common, and if treated early may be arrested in its progress towards the tendon sheath.

The late Mr. Morrant Baker drew attention to the fact that the sheath of the flexor tendons has no special proneness to be the site of the inflammation, and that it is most generally secondarily involved. The decision as to whether the sheath is actually inflamed, is often a most difficult one, but Mr. Baker's test may be of great help. If the sheath be invaded the patient will have lost the power of voluntarily flexing the terminal phalanx of the finger. Whether this has occurred or no is best determined by the surgeon placing his thumb against the palmar aspect of the second phalanx of the affected finger, and requesting the patient to endeavour to bend the last phalanx. If the flexor profundus tendon is bound down, as it probably will be if the theca be inflamed, then the ungual phalanx will remain motionless, but if there be any range of movement whatever there is presumably but little affection of the sheath.

A free incision into the infiltrated connective tissues, but so made as to carefully avoid opening into the

Fig. 1.—Arrangement of the synovial sheaths of the flexor tendons in the palm and fingers: *a*, Sheath for tendon of flexor longus pollicis; *b*, sheath for tendons of little finger, continuous with *c*, the great palmar bursa; *d*, the anterior annular ligament; *e*, sheath for index finger, ending blindly.

flexor tendon sheath if it is not involved, will speedily bring about a cessation of the inflammatory process. When, however, there is presumptive evidence that there is septic fluid within the thecal cavity, then an incision aimed to open this freely may be made. Here, again, I would depart from the usual plan of making one long median wound, and would rather proceed to evacuate the fluid by an incision, or even two, one on either side, placed rather laterally. This will avoid any chance of cutting into the tendons themselves, and further tend to prevent their protrusion through the wound. Mr. Heath and Mr. Baker both advise an incision made on the palmar aspect over the head of the metacarpal bone or thereabouts, for they say that by this wound free evacuation of pus is obtained with the minimum risk of sloughing of the tendons.

Suppuration occurring in the sheath of the tendons of the little finger is very liable to be followed by deep-seated suppuration in the palm of the hand, and the pus may even track up behind the anterior annular ligament into the forearm. The reason of this extension of the septic process is that the synovial sheath for the little finger is directly continuous with the great palmar synovial bursa. A somewhat similar result may happen when the thumb is the digit involved, for the sheath here, although not communicating with the bursa in the palm, yet runs up beside it even into the forearm. The synovial sheaths for the tendons of the index, middle, and ring fingers terminate blindly opposite the heads of their corresponding metacarpal bones. The arrangement of these sheaths is well seen in the diagram, Fig. 1.

Lastly, it must be remembered that an acute infective periostitis of one of the phalanges may be the primary affection. This condition, dependent upon the invasion of the periosteum by micro-organisms, will almost invariably lead to necrosis of the attacked phalanx. When this has happened it will be found that the wound refuses to heal, and protuberant granulations form, and the introduction of a probe elicits the roughness of dead bone. Its removal by a free incision is imperative, otherwise there will be a great tendency for the inflammation to spread to the palm.

## Figures and Tables

**Fig. 1. f1:**